# Sex-Specific Genetic Structure and Social Organization in Central Asia: Insights from a Multi-Locus Study

**DOI:** 10.1371/journal.pgen.1000200

**Published:** 2008-09-26

**Authors:** Laure Ségurel, Begoña Martínez-Cruz, Lluis Quintana-Murci, Patricia Balaresque, Myriam Georges, Tatiana Hegay, Almaz Aldashev, Firuza Nasyrova, Mark A. Jobling, Evelyne Heyer, Renaud Vitalis

**Affiliations:** 1 Muséum National d'Histoire Naturelle – Centre National de la Recherche Scientifique UMR 5145 – Université Paris 7, Éco-Anthropologie et Ethnobiologie, Musée de l'Homme, Paris, France; 2 Human Evolutionary Genetics Unit, CNRS URA3012, Institut Pasteur, Paris, France; 3 Department of Genetics, University of Leicester, Leicester, United Kingdom; 4 Uzbek Academy of Sciences, Institute of Immunology, Tashkent, Uzbekistan; 5 Institute of Molecular Biology and Medicine, National Center of Cardiology and Internal Medicine, Bishkek, Kyrgyzstan; 6 Tajik Academy of Sciences, Institute of Plant Physiology and Genetics, Dushanbe, Tajikistan; University of Chicago, United States of America

## Abstract

In the last two decades, mitochondrial DNA (mtDNA) and the non-recombining portion of the Y chromosome (NRY) have been extensively used in order to measure the maternally and paternally inherited genetic structure of human populations, and to infer sex-specific demography and history. Most studies converge towards the notion that among populations, women are genetically less structured than men. This has been mainly explained by a higher migration rate of women, due to patrilocality, a tendency for men to stay in their birthplace while women move to their husband's house. Yet, since population differentiation depends upon the product of the effective number of individuals within each deme and the migration rate among demes, differences in male and female effective numbers and sex-biased dispersal have confounding effects on the comparison of genetic structure as measured by uniparentally inherited markers. In this study, we develop a new multi-locus approach to analyze jointly autosomal and X-linked markers in order to aid the understanding of sex-specific contributions to population differentiation. We show that in patrilineal herder groups of Central Asia, in contrast to bilineal agriculturalists, the effective number of women is higher than that of men. We interpret this result, which could not be obtained by the analysis of mtDNA and NRY alone, as the consequence of the social organization of patrilineal populations, in which genetically related men (but not women) tend to cluster together. This study suggests that differences in sex-specific migration rates may not be the only cause of contrasting male and female differentiation in humans, and that differences in effective numbers do matter.

## Author Summary

Human evolutionary history has been investigated mainly through the prism of genetic variation of the Y chromosome and mitochondrial DNA. These two uniparentally inherited markers reflect the demographic history of males and females, respectively. Their contrasting patterns of genetic differentiation reveal that women are more mobile than men among populations, which might be due to specific marriage rules. However, these two markers provide only a limited understanding of the underlying demographic processes. To obtain an independent picture of sex-specific demography, we developed a new multi-locus approach based on the analysis of markers from the autosomal and X-chromosomal compartments. We applied our method to 21 human populations sampled in Central Asia, with contrasting social organizations and lifestyles. We found that, in patrilineal populations, not only the migration rate but also the number of reproductive individuals is likely to be higher for women. This result does not hold for bilineal populations, for which both the migration rate and the number of reproductive individuals can be equal for both sexes. The social organization of patrilineal populations is the likely cause of this pattern. This study suggests that differences in sex-specific migration rates may not be the only cause of contrasting male and female differentiation in humans, and that differences in effective numbers do matter.

## Introduction

Understanding the extent to which sex-specific processes shape human genetic diversity has long been a matter of great interest for human population geneticists [1],[2]. To date, as detailed in Table 1, the focus has mainly been on the analysis of uniparentally inherited markers: mitochondrial DNA (mtDNA) and the non-recombining portion of the Y chromosome (NRY). A large number of studies have found that the level of differentiation was greater for the Y chromosome than for mtDNA, both at a global [3] and a local scale [4]–[11], for a review see [12]. This result has mainly been explained by patrilocality, a widespread tendency for men to stay in their birthplace while women move to their husband's house [13] (see Table 1 for more detailed interpretations). This hypothesis of a higher migration rate of women has been especially strengthened by the comparison of patrilocal and matrilocal populations at a local scale [14]–[17]. These studies have shown that in patrilocal populations, genetic differentiation is stronger among men than among women, while the reverse is observed in matrilocal populations. It is also noteworthy that the absolute difference between male and female genetic structure is more pronounced in patrilocal than in matrilocal populations [16]. Interestingly, while social practices seem to consistently influence the sex-specific demography at a local scale, the robustness of a sex-specific genetic structure at a global scale is still a challenging issue (see Table 1). A recent analysis of mtDNA and NRY variation at a global scale, which used the same panel of populations for both categories of markers (an omission that was criticized in Seielstad et al.'s [3] study [18]) showed no difference between the male and female genetic structure [19]. Consistent with this result, an analysis of the autosomal and X-linked microsatellite markers in the HGDP-CEPH Human Genome Diversity Cell Line Panel showed no major differences between the demographic history of men and women [20]. The apparent paradox between local and global trends can be resolved though, since the geographical clustering of populations with potentially different lifestyles may minimize the differences in sex-specific demography at a global scale [21],[22]. It may also be that the global structure reflects more ancient, pre-agricultural, social patterns, as patrilocality may only have increased in human societies only with the recent transition to agriculture [12].

**Table 1:** Human sex-specific demography inferred from genetic data.

Region	Markers	Method	Social organization^a^	Differences in demographic parameters between males and females^b^			References
				Sex-biased migration		Skewed effective population size	
GLOBAL	mtDNA, NRY SNPs^c^	Genetic structure (AMOVA^d^)	NA^e^	None			[19]
GLOBAL	Autosomal STRs^f^, X-linked STRs	Genetic structure (AMOVA)	NA	None			[20]
GLOBAL	mtDNA, NRY SNPs^c^	Coalescent-based (TMRCA^g^ estimates)	NA	*m*_f_>*m*_m_ (patrilocality)	and/or	*N*_f_>*N*_m_ (polygyny)	[24]
GLOBAL^h^	mtDNA, NRY STRs+SNPs, Autosomal STRs+SNPs	Genetic structure (*F*_ST_)	NA	*m*_f_>*m*_m_ (patrilocality)		Considered as negligible^i^	[3]
GLOBAL^h^	NRY SNPs	Coalescent-based (mismatch distributions)	NA	Not considered^j^		*N*_f_>*N*_m_ (polygyny)	[23]
India	mtDNA	Genetic structure (*R*_ST_, haplotype sharing)	Endogamy, patrilocality	None			[21]
	NRY STRs		Endogamy, matrilocality	None			
Sinai peninsula	mtDNA, NRY	Genetic diversity	Endogamy and rare patrilocal exogamy, polygyny	*m*_f_>*m*_m_ (patrilocality)	and/or	*N*_f_>*N*_m_ (polygyny)	[4]
West New Guinea	mtDNA, NRY STRs+SNPs	Genetic structure and diversity (*F*_ST_, *R*_ST_, haplotype diversity)	Exogamy, patrilocality, patrilineality, polygyny	*m*_f_>*m*_m_ (patrilocality)	and/or	*N*_f_>*N*_m_ (polygyny, warfare)	[7]
Sub-Saharan Africa	mtDNA, NRY STRs+SNPs	Genetic structure (AMOVA)	FPP^k^: patrilocality, high polygyny	*m*_f_>*m*_m_ (patrilocality)	and/or	*N*_f_>*N*_m_ (polygyny)	[15]
			HGP^l^: moderate patrilocality, low polygyny	*m*_f_<*m*_m_ (multilocality)	and/or	*N*_f_<*N*_m_	
Thailand	mtDNA, NRY STRs	Coalescent-based (Approximate Bayesian Computation)	Patrilocality	*m*_f_>*m*_m_ (patrilocality)	and/or	*N*_f_>*N*_m_ (patrilocality)	[16]
			Matrilocality	*m*_f_<*m*_m_ (matrilocality)	and/or	*N*_f_<*N*_m_ (matrilocality)	
Eastern North America	mtDNA, NRY STRs+SNPs	Genetic structure (AMOVA), coalescent-based (MIGRATE^m^)	Patrilocality, patrilineality	*m*_f_>*m*_m_ (patrilocality)	and/or	*N*_f_>*N*_m_ (patrilocality)	[17]
			Matrilocality, matriliny	*m*_f_<*m*_m_ (matrilocality)	and/or	*N*_f_<*N*_m_ (matrilocality)	
Central Asia (pastoral populations)	mtDNA, NRY STRs	Genetic structure and diversity (AMOVA, *R*_ST_)	Exogamy, patrilineality	*m*_f_>*m*_m_ (patrilineality, exogamy)	and/or	*N*_f_>*N*_m_ (patrilineality,VRS^n^)	[11]
New Britain	mtDNA, NRY SNPs, X-linked loci	Coalescent-based (θ^o^ and TMRCA estimates)	No strong endogamy, ambilocality, polygyny	*m*_f_<*m*_m_	and	*N*_f_>*N*_m_ (polygyny)	[25]
Central Asia	mtDNA, NRY STRs	Genetic structure (AMOVA)	Exogamy, patrilocality, polygyny	*m*_f_>*m*_m_ (patrilocality)		Considered as negligible	[5]
Thailand	mtDNA, NRY STRs	Genetic structure and diversity (haplotype diversity, *R*_ST_)	Patrilocality	*m*_f_>*m*_m_ (patrilocality)		Considered as negligible	[14]
			Matrilocality	*m*_f_<*m*_m_ (matrilocality)		Considered as negligible	
Sub-Saharan Africa^h^	mtDNA, NRY SNPs	Genetic structure and diversity (haplotype diversity, AMOVA)	NA^c^	*m*_f_<*m*_m_		Not considered	[22]
Continental Asia^h^	mtDNA, NRY SNPs	Genetic structure (*F*_ST_)	NA^c^	*m*_f_>*m*_m_ (patrilocality)		Not considered	[6]
Russia	mtDNA, NRY SNPs	Genetic structure (*F*_ST_)	Patrilocality, patrilineality	*m*_f_>*m*_m_ (patrilocality)		Not considered	[8]
Caucasus	mtDNA, NRY SNPs	Genetic structure (AMOVA)	NA	*m*_f_>*m*_m_ (patrilocality)		Not considered	[9]
Turkey	mtDNA, NRY STRs+SNPs	Genetic structure (AMOVA)	NA	*m*_f_>*m*_m_ (patrilocality)		Not considered	[10]

This table summarizes the observed patterns of sex-specific differences in demographic parameters reported in a number of recent studies. The first column lists the location of the sampled populations, or indicates whether the study is conducted at a global scale. The second column gives the markers used, and the third column indicates the statistical methods employed. The fourth column provides indications on social organization, available a priori for the populations under study. In the fifth and sixth columns, the authors' interpretations of sex-specific differences in demographic parameters are given, with respect to skewed gene flow and/or effective numbers.

a Indications on social organization, marriage rules, etc., as provided by the authors.

b The differences in demographic parameters between males and females, as inferred by the authors, are given in terms of sex-biased gene flow, and skewed effective numbers; the authors' interpretation to the observed pattern is given in parentheses, when available.

c Single nucleotide polymorphisms.

d Analysis of molecular variance [69].

e Not available (no detailed information given by the authors concerning social organization, marriage rules, etc.).

f Short tandem repeats.

g Time to the most recent common ancestor.

h mtDNA and NRY were not sampled in the same individuals or populations.

i The authors discussed a possible difference in demographic parameters between males and females, but considered it as negligible.

j The authors did not consider this pattern.

k Food-producer populations.

l Hunter-gatherer populations.

m Monte Carlo Markov chain method to estimate population sizes and migration rates [70].

n Variance in Reproductive Success.

o population-mutation parameter.

The higher differentiation level found on the NRY as compared to mtDNA at a local scale could also be the consequence of a higher effective number of women, for example through the practice of polygyny, a tendency for men (but not for women) to have multiple mates [4], [7], [15], [23]–[25], and/or through the paternal transmission of reproductive success [11]. However, the influence of such processes on genetic structure has often been considered as negligible, since realistic rates of polygyny cannot create large differences in male and female genetic structure [3],[5],[14]. Hence, until now, the effect of local social processes on male and female effective numbers has not been investigated directly, possibly because current methods fail to unravel the relative contribution of effective number and migration rate on the differentiation level [26]. The consequence is that the vast majority of studies fail to show whether the observed differentiation arises from sex-specific differences in migration rate, effective numbers, or both (see Table 1). New methods need therefore to be developed in order to appreciate the relative influence of sex-biased dispersal and differences in effective numbers on genetic structure.

Another limitation to the use of uniparentally inherited markers stems from the fact that each of them is, in effect, a single genetic locus. For that reason, we cannot test for the robustness of the sex-specific genetic structure on these markers. We cannot either rule out the possibility that mtDNA and NRY, which contain multiple linked genes, may be shaped by selection [27],[28]. This raises the question of whether results based on uniparentally inherited markers simply reflect stochastic variation, or real differences in sex-specific demography. To answer this question, we propose a novel approach based on the joint analysis of autosomal and X-linked markers. This multi-locus analysis has the potential of providing more robust information, as these markers give an independent picture of sex-specific demography. This approach also aims to disentangle the effects of sex-biased dispersal and effective numbers on genetic structure.

**Figure 1:**
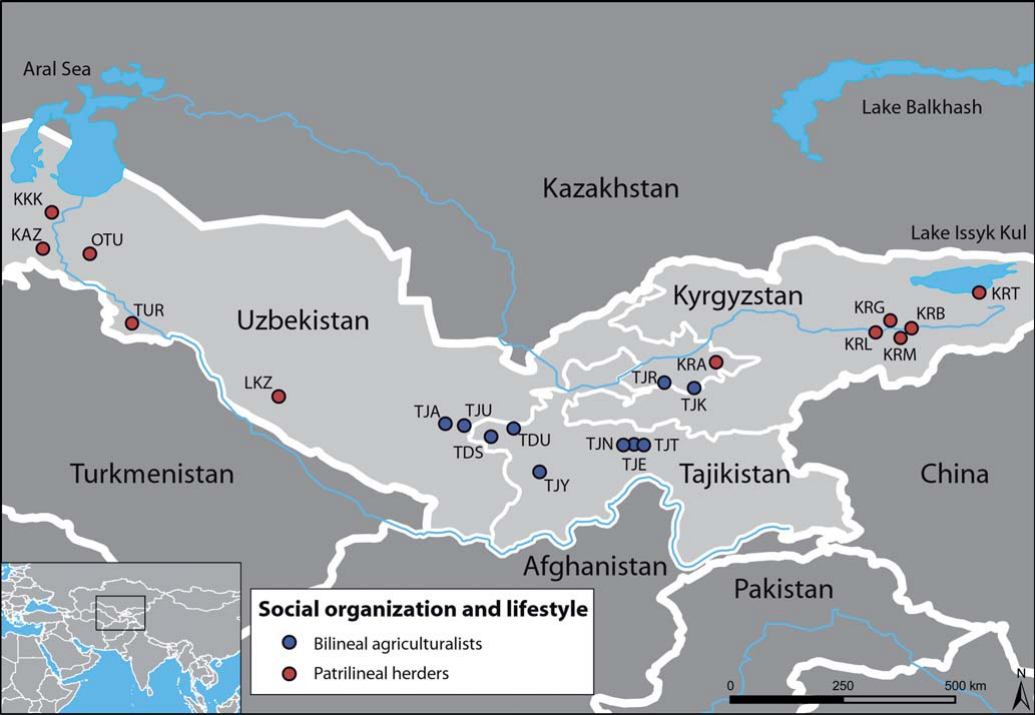
Geographic map of the sampled area, with the 21 populations studied. Bilineal agriculturalist populations are in blue (Tajiks); Patrilineal herders with a semi-nomadic lifestyle are in red (Kazaks, Karakalpaks, Kyrgyz and Turkmen).

In order to recognize the impact of social organization on these differences, we investigate sex-specific genetic structure in human populations of Central Asia (Figure 1), where various ethnic groups, characterized by different languages, lifestyles and social organizations, co-exist. Although all groups share a patrilocal organization, Tajiks (sedentary agriculturalists) are bilineal, i.e. they are organized into nuclear or extended families where blood links and rights of inheritance through both male and female ancestors are of equal importance, and they preferentially establish endogamous marriages with cousins. By contrast, Kazaks, Karakalpaks, Kyrgyz and Turkmen (traditionally nomadic herders) are patrilineal, i.e. they are organized into paternal descent groups (tribes, clans, lineages), and they practice exogamous marriages, in which a man chooses a bride from a different clan.

## Results/Discussion

### Uniparentally-Inherited Markers

We sampled 780 healthy adult men from 10 populations of bilineal agriculturalists and 11 populations of patrilineal herders from West Uzbekistan to East Kyrgyzstan, representing 5 ethnic groups (Tajiks, Kyrgyz, Karakalpaks, Kazaks, and Turkmen) (see Figure 1 and Table 2). We genotyped all bilineal populations, and 8 out of 11 patrilineal populations at the HVS-I locus of mtDNA, and at 11 microsatellite markers on the NRY (for more details on the markers used, see Table 3). The overall genetic differentiation was higher for NRY, as compared to mtDNA, both among the 10 bilineal agriculturalist populations 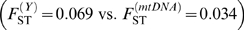, and among the subset of 8 patrilineal herder populations 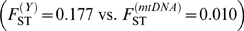. Assuming an island model of population structure, this implies that female migration rate (*m*_f_), and/or the effective number of females (*N*_f_), is higher than of the corresponding parameters for males (*m*_m_ and *N*_m_). These results also suggest that the differences in sex-specific genetic structure are much more pronounced in the patrilineal herders than in the bilineal agriculturalists. From the above *F*_ST_ estimates, we obtained the female-to-male ratio of the effective number of migrants per generation (see the Methods section for details): *N*_f_ *m*_f_/*N*_m_ *m*_m_≈2.1 for bilineal populations and *N*_f_ *m*_f_/*N*_m_ *m*_m_≈21.6 for patrilineal populations. The ratio in patrilineal populations is thus one order of magnitude higher than in bilineal populations. However, since each of these markers is a single genetic locus, we cannot test for the robustness of the sex-specific genetic structure on these markers. We therefore examined the amount of information contained in multi-locus data on autosomal and X-linked markers, both of which average over male and female histories.

**Table 2:** Sample description.

Sampled populations (area)	Acronym	Location	Long.	Lat.	*n*_X_	*n*_A_	*n*_Y_	*n*_mt_
**Bilineal agriculturalists**								
Tajiks (Samarkand)	TJA	Uzbekistan/Tajikistan border	**39.54**	**66.89**	26	31	32	32
Tajiks (Samarkand)	TJU	Uzbekistan/Tajikistan border	**39.5**	**67.27**	27	29	29	29
Tajiks (Ferghana)	TJR	Tajikistan/Kyrgyzstan border	**40.36**	**71.28**	30	29	29	29
Tajiks (Ferghana)	TJK	Tajikistan/Kyrgyzstan border	**40.25**	**71.87**	26	26	35	40
Tajiks (Gharm)	TJE	Northern Tajikistan	**39.12**	**70.67**	29	25	27	31
Tajiks (Gharm)	TJN	Western Tajikistan	**38.09**	**68.81**	33	24	30	35
Tajiks (Gharm)	TJT	Northern Tajikistan	**39.11**	**70.86**	31	25	30	32
Tajiks (Penjinkent)	TDS	Uzbekistan/Tajikistan border	**39.28**	**67.81**	30	25	31	31
Tajiks (Penjinkent)	TDU	Uzbekistan/Tajikistan border	**39.44**	**68.26**	40	25	31	40
Tajiks (Yagnobs from Douchambe)	TJY	Western Tajikistan	**38.57**	**68.78**	39	25	36	40
**Patrilineal herders with a semi-nomadic lifestyle**								
Karakalpaks (Qongrat from Karakalpakia)	KKK	Western Uzbekistan	**43.77**	**59.02**	56	45	54	55
Karakalpaks (On Tört Uruw from Karakalpakia)	OTU	Western Uzbekistan	**42.94**	**59.78**	49	45	54	53
Kazaks (Karakalpakia)	KAZ	Western Uzbekistan	**43.04**	**58.84**	47	49	50	50
Kazaks (Bukara)	LKZ	Southern Uzbekistan	**40.08**	**63.56**	20	25	20	31
Kyrgyz (Andijan)	KRA	Tajikistan/Kyrgyzstan border	**40.77**	**72.31**	31	45	46	48
Kyrgyz (Narin)	KRG	Middle Kyrgyzstan	**41.6**	**75.8**	20	18	20	20
Kyrgyz (Narin)	KRM	Middle Kyrgyzstan	**41.45**	**76.22**	21	21	22	26
Kyrgyz (Narin)	KRL	Middle Kyrgyzstan	**41.36**	**75.5**	36	22	-	-
Kyrgyz (Narin)	KRB	Middle Kyrgyzstan	**41.25**	**76**	31	24	-	-
Kyrgyz (Issyk Kul)	KRT	Eastern Kyrgyzstan	**42.16**	**77.57**	33	37	-	-
Turkmen (Karakalpakia)	TUR	Western Uzbekistan	**41.55**	**60.63**	42	47	51	51

Long., longitude; Lat., latitude. *n*_X_, *n*_A_, *n*_Y_ and *n*_mt_: sample size for X-linked, autosomal, Y-linked and mitochondrial markers, respectively.

**Table 3:** Level of diversity and differentiation for NRY markers and mtDNA.

NRY markers			*F*_ST_	
Locus name	Allelic richness (AR)	*H*_e_	Herders	Agriculturalists
DYS426	4	0.500	0.3326	0.0068
DYS393	8	0.492	0.1095	0.0517
DYS390	8	0.739	0.1229	0.1253
DYS385 a/b	15	0.858	0.1414	0.0278
DYS388	9	0.531	0.3003	0.0736
DYS19	7	0.743	0.1081	0.1310
DYS392	10	0.516	0.1345	0.0701
DYS391	7	0.495	0.2533	0.0686
DYS389I	6	0.541	0.1537	0.1395
DYS439	7	0.725	0.1638	0.0291
DYS389II	8	0.763	0.1556	0.0395

mtDNA			*F*_ST_	
Locus name	Polymorphic sites	*H*_e_	Herders	Agriculturalists
HVS-I	121	0.0156	0.0098	0.0343

We calculated the total allelic richness (*AR*) (over all populations) and the expected heterozygosity *H*_e_ [55] using Arlequin version 3.1 [56]. Genetic differentiation among populations was measured both per locus and overall loci, using Weir and Cockerham's *F*_ST_ estimator [57], as calculated in Genepop 4.0 [58]. We calculated the total number of polymorphic sites, the unbiased estimate of expected heterozygosity *H*_e_ [55], and *F*_ST_ using Arlequin version 3.1 [56].

### A New Multi-Locus Approach

**Figure 2:**
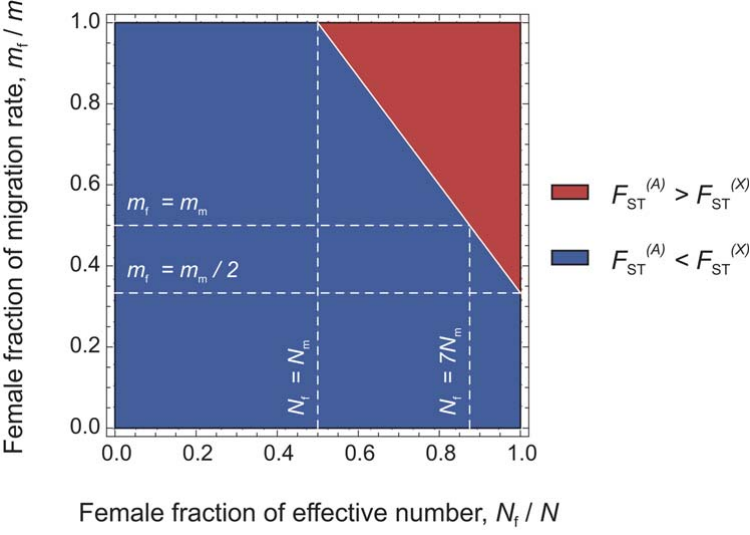
Diagram representing the relative values of expected genetic differentiation for autosomal markers 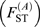 and for X-linked markers 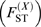. In the red upper right triangle, the *F*_ST_ estimates for autosomal markers are higher than for X-linked markers. In this case, *N*_f_/*N* is necessarily larger than 0.5. In the blue region of the figure, the *F*_ST_ estimates for autosomal markers are lower than for X-linked markers. The white plain line, at which 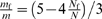, represents the set of (*N*_f_/*N*, *m*_f_/*m*) values where the autosomal and X-linked *F*_ST_ estimates are equal. In this case 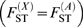, if *N*_f_ = *N*_m_, then the lower effective size of X-linked markers (which would be three-quarters that of autosomal markers) can only be balanced by a complete female-bias in dispersal (*m*_f_/*m* = 1). Conversely, if *m*_f_ = *m*_m_, the large female fraction of effective numbers compensates exactly the low effective size of X-linked markers only for *N*_f_ = 7*N*_m_. Last, if *m*_f_ = *m*_m_/2, then the autosomal and X-linked *F*_ST_ estimates can only be equal as the number of males tends towards zero.

In the infinite island model of population structure with two classes of individuals (males and females), we obtained the following expressions of *F*_ST_ (see the Methods section for details):
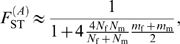
(1)for autosomal genes, and
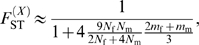
(2)for X-linked genes. A special case of interest occurs when 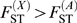, i.e. when the differentiation of X-linked genes exactly equals that of autosomal genes. Combining eqs (1) and (2), we find that this occurs for 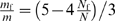, with *N* = *N*_f_+*N*_m_ and *m* = *m*_f_+*m*_m_. Furthermore, as shown in Figure 2, if we observe a lower genetic differentiation of autosomal markers, as compared to X-linked markers (blue zone in Figure 2), this suggests that 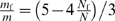. This may happen, e.g., for *N*_f_ = *N*_m_ and *m*_f_ = *m*_m_, i.e. for equal effective numbers of males and females and unbiased dispersal. But if autosomal markers are more differentiated than X-linked markers (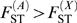, see the red upper-right triangle in Figure 2), this implies that 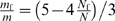. In this case, since *m*_f_/*m* and *N*_f_/*N* are ratios varying between 0 and 1, the effective number of females must be higher than that of males (*N*_f_>*N*_m_), and the female migration rate must be higher than half the male migration rate (*m*_f_>*m*_m_/2). Hence, a prediction from this model is that when 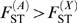, the effective number of females is higher than that of males, whatever the pattern of sex-specific dispersal. This suggests that it is indeed possible to test for differences in effective numbers between males and females from the joint analysis of autosomal and X-linked data. We note however that when 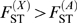, we cannot conclude on the relative male and female effective numbers and migration rates.

We tested the above prediction in the 10 bilineal agriculturalist populations and 11 patrilineal herder populations sampled in Central Asia by comparing the genetic structure estimated from 27 unlinked polymorphic autosomal microsatellite markers (*AR* = 16.2, *H*_e_ = 0.803 on average) to that from 9 unlinked polymorphic X-linked microsatellite markers (*AR* = 12.6, *H*_e_ = 0.752 on average) (for more details on the markers used, see Table 4). Overall heterozygosity was not significantly different between X-linked and autosomal markers, neither in the pooled sample (two-tailed Wilcoxon sum rank test; *p* = 0.09), nor in the bilineal agriculturalists (*p* = 0.13) or the patrilineal herders (*p* = 0.12). The overall population structure was significantly higher for autosomal as compared to X-linked markers among patrilineal herders: 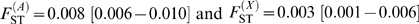 (one-tailed Wilcoxon sum rank test; 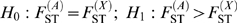; *p* = 0.02). Among bilineal agriculturalists, the result was not significant: 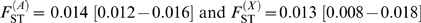 (*p* = 0.36). From these results, and following our model predictions, we conclude that in patrilineal herders (where 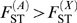), the effective number of females is higher than that of males. This conclusion does not hold for the bilineal agriculturalists.

**Table 4:** Level of diversity and differentiation for X-linked and autosomal markers.

			*F*_ST_	
Locus name	Allelic richness (AR)	*H*_e_	Herders	Agriculturalists
**X-linked markers**				
CTAT014	19	0.746	0.0018	0.0225
GATA124E07	15	0.847	0.0024	0.0136
GATA31D10	8	0.697	0.0069	0.0007
ATA28C05	7	0.722	0.0086	0.0179
AFM150xf10	14	0.832	−0.0021	0.0152
GATA100G03	14	0.734	−0.0019	0.0084
AGAT121P	15	0.593	−0.0016	0.0048
ATCT003	10	0.797	0.0095	0.0261
GATA31F01	11	0.804	0.0069	0.0053
**Autosomal markers**				
AFM249XC5	19	0.848	0.0080	0.0081
ATA10H11	13	0.680	0.0128	0.0193
AFM254VE1	14	0.837	0.0105	0.0086
AFMA218YB5	14	0.852	0.0030	0.0151
GGAA7G08	22	0.896	0.0096	0.0138
GATA11H10	16	0.776	0.0017	0.0056
GATA12A07	16	0.857	0.0001	0.0163
GATA193A07	15	0.825	0.0064	0.0087
AFMB002ZF1	11	0.820	0.0028	0.0169
AFMB303ZG9	16	0.858	0.0090	0.0148
ATA34G06	12	0.675	0.0088	0.0132
GATA72G09	18	0.884	−0.0023	0.0131
GATA22F11	21	0.897	0.0152	0.0144
GGAA6D03	13	0.831	0.0048	0.0176
GATA88H02	17	0.892	0.0063	0.0056
SE30	15	0.762	0.0084	0.0103
GATA43C11	16	0.870	0.0028	0.0093
AFM203YG9	14	0.753	0.0105	0.0084
AFM157XG3	13	0.753	0.0147	0.0196
UT2095	16	0.738	0.0032	0.0112
GATA28D01	25	0.896	0.0156	0.0139
GGAA4B09	19	0.707	0.0034	0.0208
ATA3A07	12	0.746	0.0078	0.0070
AFM193XH4	11	0.716	0.0164	0.0129
GATA11B12	26	0.896	0.0104	0.0265
AFM165XC11	13	0.785	0.0058	0.0185
AFM248VC5	20	0.620	0.0246	0.0145

We calculated the allelic richness (*AR*) and unbiased estimates of expected heterozygosity *H*_e_ [55], obtained both by locus and on average with Arlequin version 3.1 [56]. Genetic differentiation among populations was measured both per locus and overall loci, using Weir and Cockerham's *F*_ST_ estimator [57] as calculated in Genepop 4.0 [58].

From our model, it is possible to get more precise indications on the sets of (*N*_f_/*N*, *m*_f_/*m*) values that are compatible with our data. Rearranging eqs (1–2), we get:
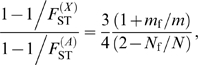
(3)i.e.:
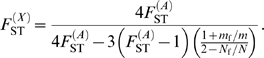
(4)


**Figure 3:**
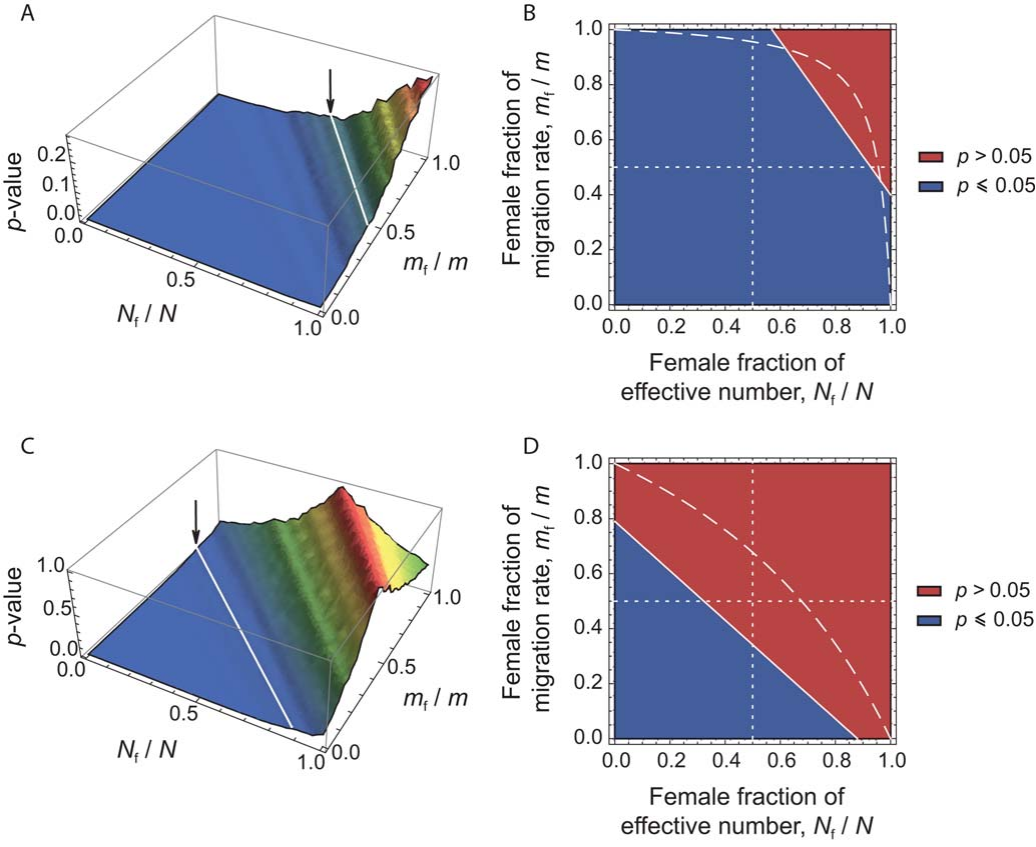
*p*-values of Wilcoxon tests plotted in the (*N*_f_/*N*, *m*_f_/*m*) parameter space. For each set of (*N*_f_/*N*, *m*_f_/*m*) values, we applied the transformation in eq. (4), and tested whether our data on autosomal and X-linked markers were consistent, given the hypothesis defined by the set of (*N*_f_/*N*, *m*_f_/*m*) values. (A) Surface plot of the *p*-values, as a function of the female fraction of effective number and the female fraction of migration rate, for the herders (11 populations). The arrow indicates the line that separates the region where *p*≤0.05 from that where *p*>0.05. Non-significant *p*-values (*p*>0.05) correspond to the values of (*N*_f_/*N*, *m*_f_/*m*) that could not be rejected, given our data. (B) Contour plots, for the same data. The dashed line indicates the range of (*N*_f_/*N*, *m*_f_/*m*) values inferred from the ratio of NRY and mtDNA population structure, as obtained from the relationship: 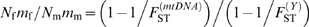. The dotted lines correspond to the cases where *N*_f_ = *N*_m_ (vertical line) and *m*_f_ = *m*_m_ (horizontal line). (C) and (D) as (A) and (B), respectively, for the agriculturalists (10 populations).

For any given set of (*N*_f_/*N*, *m*_f_/*m*) values, we can therefore calculate from eq. (4) the expected value of 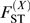 for each 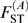 estimate in the dataset. We can then test the null hypothesis 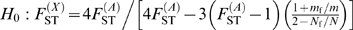 by comparing the distribution of observed and expected 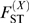 values. If the hypothesis can be rejected at the *α* = 0.05 level, then the corresponding set of (*N*_f_/*N*, *m*_f_/*m*) values can also be rejected. Following Ramachandran et al. [20], we varied the values of the ratios *N*_f_/*N* and *m*_f_/*m* (respectively, the female fraction of effective number, and the female fraction of the total migration rate) from 0 to 1, with an interval of 0.01 between consecutive values. For each set of (*N*_f_/*N*, *m*_f_/*m*) values, we applied the transformation in eq. (4) to each of the 27 locus-specific 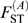 values observed. Thus, for each set of (*N*_f_/*N*, *m*_f_/*m*) values, we obtained 27 expected values of 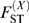, given our data. These expected values of 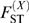 were then compared to the 9 observed locus-specific 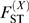 in our dataset, and we calculated the *p*-value for a two-sided Wilcoxon sum rank test between the list of 27 expected 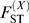 values and the 9 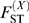 observed in the dataset. The results are depicted in Figure 3. Significant *p*-values (*p*≤0.05) correspond to a significant difference between the observed and expected values, thus to sets of (*N*_f_/*N*, *m*_f_/*m*) values that are rejected, given our data (see the blue region in Figure 3). Conversely, non-significant *p*-values (*p*>0.05) correspond to sets of (*N*_f_/*N*, *m*_f_/*m*) values that cannot be rejected (see the red region in Figure 3).

For the patrilineal herder populations (Figures 3A–3B), most sets of (*N*_f_/*N*, *m*_f_/*m*) values are rejected, except those corresponding to larger effective numbers for females (from Figures 3A–3B: *N*_f_/*N*>0.55, i.e. *N*_f_>1.27*N*_m_) and *m*_f_>0.67*m*_m_. Because the multi-locus estimate of 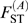 is significantly higher than the estimate of 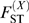, we expected to find such patterns of non-significant values (see Figure 2). For the bilineal agriculturalist populations, we could not reject the hypothesis that the effective numbers and migration rates are equal across males and females or even lower in females (see Figures 3C–3D). This is also reflected by the fact that the estimates of 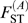 were not significantly higher than the estimates of 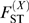 in those populations.

Finally, we have shown that the effective number of women is higher than that of men among patrilineal herders, but not necessarily among bilineal agriculturalists. Furthermore, a close inspection of the results depicted in Figures 3A and 3B reveals that, among herders, we reject all the sets of (*N*_f_/*N*, *m*_f_/*m*) values for which *m*_f_<*m*_m_ at the *α* = 0.10 level. This is not true for agriculturalists. This suggests that the migration rates are also likely to be higher for women than for men in patrilineal populations, as compared to bilineal populations (compare Figures 3B and 3D). Although both groups are patrilocal, such a difference in sex-specific migration patterns might be expected, since patrilineal herders are exogamous (among clans) and bilineal agriculturalists are preferentially endogamous. For example, it was observed that in patrilocal and matrilocal Indian populations, where migrations are strictly confined within endogamous groups, sex-specific patterns were not influenced by post-marital residence [21].

### What Could Explain a Larger Effective Number of Females?

While an influence of post-marital residence on the migration rate of women and men has already been widely proposed [14]–[17] (see also Table 1), the factors that may locally affect the effective number of women, relatively to that of men, are not well recognized. As seen in Table 1, although a number of studies have compared matrilocal and patrilocal populations, few have compared contrasting groups of populations with respect to other factors as, e.g., the tendency for polygyny [15]. Furthermore, a number of these studies lack ethnological information a priori, concerning social organization, marriage rules, etc., which makes interpretation somewhat difficult (see Table 1). Here, we compared two groups of patrilocal populations with contrasting social organizations, and at least five non-mutually exclusive interpretations for a larger effective number of females can be invoked:

- *Social organization*, i.e. the way children are affiliated to their parents, can deeply affect sex-specific genetic variation. In Central Asia, herder populations are organized in patrilineal descent groups (tribes, clans, lineages). This implies that children are systematically affiliated with the descent groups of the father. Chaix et al. [11] showed that the average number of individuals carrying the same Y chromosome haplotype was much higher in patrilineal herder populations than in bilineal agriculturalist populations (where children are affiliated both to the mother and the father). These “identity cores” would be the direct consequence of the internal dynamics of their patrilineal organization. Indeed, the descent groups are not formed randomly and related men tend to cluster together, e.g. through the recurrent lineal fission of one population into new groups. This particular dynamics increases relatedness among men, and may therefore reduce the effective number of men, as compared to women.
- Indirectly, the social organization can also deflate the effective number of men through *the transmission of reproductive success* [29] if this success is culturally transmitted exclusively from fathers to sons. Because herders are patrilineal (so that inheritance is organized along paternal descent groups), social behaviors are more likely to be inherited through the paternal line of descent only. It has recently been argued that the rapid spread of Genghis Khan's patrilineal descendants throughout Central Asia was explained by this social selection phenomenon [30]. The correlation of fertility through the patriline has also been described in patrilineal tribes in South America [31]. By contrast, in bilineal societies such as the agriculturalists of Central Asia, social behaviors that influence reproductive success are more likely to be transmitted by both sexes. Furthermore, differences of cultural transmission of fitness between hunter-gatherers and agriculturalists have already been reported [32]. Interestingly, a slightly higher matrilineal intergenerational correlation in offspring number has been observed in the Icelandic population, which suggests that in some populations, reproductive behaviors can be maternally-inherited [33].
- *Polygyny*, in which the husband may have multiple wives, has often been invoked as a factor that could reduce the effective number of men [4], [7], [15], [23]–[25]. While we could not find any evidence of polygyny in present-day Central Asian populations, this custom was traditionally practiced in the nomadic herder Kazak populations, although limited to the top 10 percent of men from the highest social rank [5],[34]. Hence, even though we lack ethnological data to determine to what extent herders are or were practicing polygyny in a recent past, the practice of polygyny among herders in Central Asia might have influenced (at least partially) the observed differences in men and women effective numbers.
- *Recurrent bottlenecks in men* due to a higher pre-reproductive mortality could also severely reduce the effective numbers of men. From the study of several groups in West Papua and Papua New Guinea [7],[35], it appears that warfare may indeed lead to the quasi-extinction of adult men in some communities, while the mass killing of adult women is far more rarely reported. However, this differential mortality could also be balanced by potentially high death rates of women during childbirth. In any case, a differential mortality is equally likely to arise in herder and agriculturalist populations. It may therefore not be relevant in explaining why we detect higher effective numbers of women (as compared to men) in patrilineal herders and not in bilineal agriculturalists.
- Since our approach implicitly assumes equal male and female generation time, the observed higher effective number of women, relatively to that of men, could result from a *shorter generation time for women*, due to the tendency of women to reproduce earlier in life than men and the ability of men to reproduce at a later age than women. This has indeed been described in a number of populations with different lifestyles, from complete genealogical records or mean-age-at-first-marriage databases [33],[36],[37]. It has even been proposed to be a nearly universal trait in humans, although its magnitude varies across regions and cultures [37]. Tang et al. [38] suggested that accounting for longer generation time in males could minimize the difference between maternal and paternal demography. However, the differences in sex-specific generation times that have been reported (e.g., 28 years for the matrilines and 31 years for the patrilines in Iceland [33], 29 years for the matrilines and 35 years for the patrilines in Quebec [36]) are unlikely to explain the observed differences in male and female effective numbers [24].

### Limits of the Approach

There might also be non-biological explanations of our results, however, as they are based on the simplifying assumptions of Wright's infinite island model of population structure [39]. This model assumes (*i*) that there is no selection and that mutation is negligible, (*ii*) that each population has the same size, and sends and receives a constant fraction of its individuals to or from a common migrant pool each generation (so that geographical structure is absent), and (*iii*) that equilibrium is reached between migration, mutation and drift. On the first point, we did not find any evidence of selection, for any marker, based on Beaumont and Nichols' method [40] for detecting selected markers from the analysis of the null distribution generated by a coalescent-based simulation model (data not shown). As for the second point, we tested for the significance of the correlation between the pairwise *F*_ST_/(1−*F*_ST_) estimates and the natural logarithm of their geographical distances [41]. We found no evidence for isolation by distance, either for X-linked markers (*p* = 0.47 for agriculturalists, *p* = 0.24 for herders), or for autosomal markers (*p* = 0.92 for agriculturalists, *p* = 0.45 for herders). As for the third point, the X-to-autosomes (X/A) effective size ratio can significantly deviate from the expected three-quarters (assuming equal effective numbers of men and women) following a bottleneck or an expansion [42]. This is because X-linked genes have a smaller effective size, and hence reach equilibrium more rapidly. After a reduction of population size, the X/A diversity ratio is lower than expected, while after an expansion, the diversity of X-linked genes recovers faster than on the autosomes, and the X/A diversity ratio is then closer to unity. In the latter case, 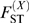 would be reduced and could then tend towards 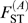. However, neither reduction nor expansion should lead to 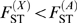, as we found in herder populations of Central Asia. Therefore, we do not expect the limits of Wright's island model to undermine our approach.

### Evaluation by Means of Stochastic Simulations

**Figure 4:**
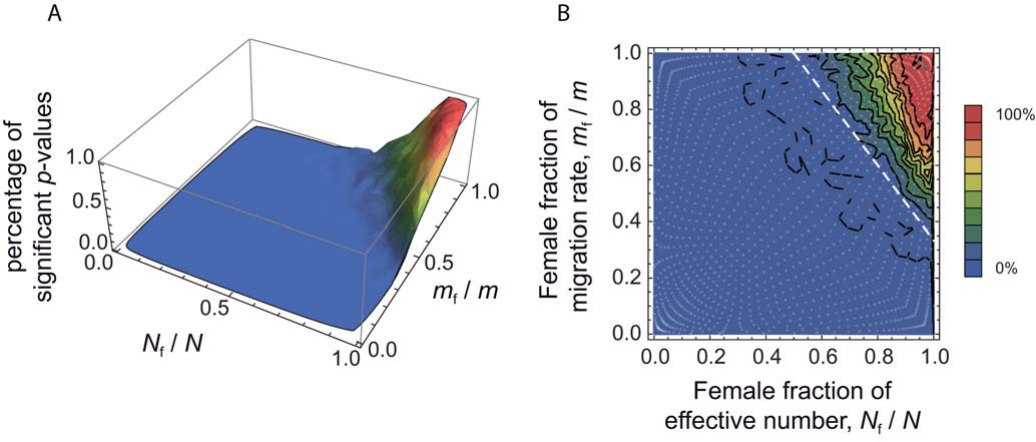
Percentage of significant tests in the (*N*_f_/*N*, *m*_f_/*m*) parameter space, for simulated data. We chose a range of 49 (*N*_f_ *m*_f_/*N*_m_ *m*_m_) ratios, varying from 0.0004 to 2401, and for each of these ratios we chose 29 sets of (*N*_f_/*N*, *m*_f_/*m*) values. By doing this, we obtained 1421 sets of (*N*_f_/*N*, *m*_f_/*m*) values, represented as white dots in the right-hand side panel B, covering the whole parameter space. For each set, we simulated 100 independent datasets using a coalescent-based algorithm, and taking the same number of individuals and the same number of loci for each genetic system as in the observed data. For each dataset, we calculated the *p*-value for a one-sided Wilcoxon sum rank test 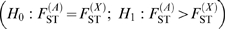, and for each set of (*N*_f_/*N*, *m*_f_/*m*) values we calculated the percentage of significant *p*-values (at the *α* = 0.05 level). A. Surface plot of the proportion of significant *p*-values (at the *α* = 0.05 level), as a function of the female fraction of effective number and the female fraction of migration rate. B. Contour plot, for the same data. The dotted line, at which 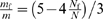, represents the set of (*N*_f_/*N*, *m*_f_/*m*) values where the autosomal and X-linked *F*_ST_'s are equal. The theory predicts that we should only find 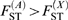 in the upper-right triangle defined by the dotted line. Hence, the proportion of significant *p*-values for any set of (*N*_f_/*N*, *m*_f_/*m*) values in this upper right triangle gives an indication of the power of the method.

We aimed to investigate to what extent the approach proposed here is able to detect differences in male and female effective numbers. To do this, we performed coalescent simulations in a finite island model, for a wide range of (*N*_f_/*N*, *m*_f_/*m*) values. The simulation parameters were set to match those of our dataset: 11 sampled demes, 30 males genotyped at 27 autosomal and 9 X-linked markers per deme (for further details concerning the simulations, see the Methods section). We used 1421 sets of (*N*_f_/*N*, *m*_f_/*m*) values, covering the whole parameter space (represented as white dots in Figure 4B). For each set of (*N*_f_/*N*, *m*_f_/*m*) parameter values, we simulated 100 independent datasets. For each dataset, we calculated the estimates of 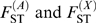 at all loci, and we calculated the *p*-value for a one-sided Wilcoxon sum rank test for the list of 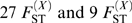 estimates 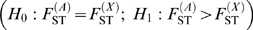. Hence, for each set of (*N*_f_/*N*, *m*_f_/*m*) parameter values, we could calculate the proportion of significant tests at the *α* = 0.05 level, among the 100 independent datasets. Figure 4 shows the distribution of the percentage of significant tests in the (*N*_f_/*N*, *m*_f_/*m*) parameter space. Theory predicts that in the upper-right triangle where 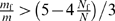, we should have 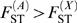. One can see from Figure 4 that, given the simulation parameters used, the method is conservative: the proportion of significant tests at the *α* = 0.05 level is null outside of the upper-right triangle. However, we find a fairly large proportion of significant tests for large *N*_f_/*N* and *m*_f_/*m* ratios which indicates (*i*) that the method presented here has the potential to detect differences in male and female effective numbers, but (*ii*) that only strong differences might be detected, for similarly sized datasets as the one considered here.

### Robustness to the Sampling Scheme

We also aimed to investigate whether the results obtained here were robust to our sampling scheme, and that our results were not biased by the inclusion of particular populations. To do this, we re-analyzed both the bilineal agriculturalists and the patrilineal herders datasets, removing one population at a time in each group. For each of these jackknifed datasets, we calculated the *p*-value of a one-sided Wilcoxon sum rank test 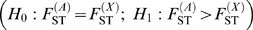, as done on the full datasets. The results are given in Table 5. We found no significant test for any of the bilineal agriculturalist groupings (*p*>0.109), which supports the idea that, in those populations, both the migration rate and the number of reproductive individuals can be equal for both sexes. In patrilineal herders, the tests were significant at the *α* = 0.05 level for 8 out of 11 population groupings. For the 3 other groupings, the *p*-values were 0.068, 0.078 and 0.073 (see Table 5). Overall, the ratio of 
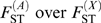
 multi-locus estimates ranged from 1.7 to 3.5 in patrilineal herders (and from 0.9 to 1.2 in bilineal agriculturalists). Although in some particular groupings of patrilineal herder populations, the difference in the distributions of 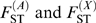 may not be strong enough to be significant, we can clearly distinguish the pattern of differentiation for autosomal and X-linked markers in patrilineal and bilineal groups. Results from coalescent simulations (see above) suggest that this lack of statistical power might be expected for 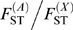 ratios close to unity. Indeed, we found that the tests were more likely to be significant for fairly large *N*_f_/*N* and *m*_f_/*m* ratios (the upper-right red region in Figure 4) which would correspond to 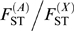 ratios much greater than one.

**Table 5:** Autosomal and X-linked differentiation on jackknifed samples.

Sample removed	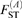	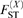	*p*-value	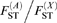
**Patrilineal groups**				
KAZ	0.0084	0.0050	0.068	1.7
KKK	0.0085	0.0050	0.078	1.7
KRA	0.0078	0.0027	0.022	2.9
KRB	0.0080	0.0030	0.028	2.7
KRG	0.0078	0.0035	0.037	2.2
KRL	0.0086	0.0038	0.018	2.3
KRM	0.0069	0.0023	0.018	3.0
KRT	0.0081	0.0044	0.047	1.8
LKZ	0.0088	0.0025	0.002	3.5
OTU	0.0089	0.0038	0.022	2.3
TUR	0.0054	0.0025	0.073	2.2
**Bilineal groups**				
TDS	0.0125	0.0109	0.443	1.1
TDU	0.0132	0.0153	0.705	0.9
TJA	0.0144	0.0123	0.109	1.2
TJE	0.0140	0.0133	0.148	1.1
TJK	0.0134	0.0131	0.457	1.0
TJN	0.0148	0.0144	0.387	1.0
TJR	0.0140	0.0141	0.401	1.0
TJT	0.0139	0.0121	0.225	1.1
TJU	0.0139	0.0127	0.283	1.1
TJY	0.0139	0.0116	0.259	1.2

For each group, we removed one sample in turn and calculated the differentiation on autosomal and X-linked markers. The *p*-value gives the result of a one-sided Wilcoxon sum rank test 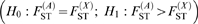, as performed on the full dataset.

### Comparison with Uniparentally-Inherited Markers

Importantly, our results on X-linked and autosomal markers are consistent with those obtained from NRY and mtDNA (see Figures 3B–3D): in these figures, the dashed line gives all the sets of (*N*_f_/*N*, *m*_f_/*m*) values that are compatible with the observed 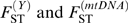 estimates. These are the sets of values that satisfy 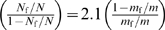 for the bilineal populations, and 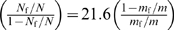 for the patrilineal populations, since we inferred *N*_f_ *m*_f_/*N*_m_ *m*_m_≈2.1 and *N*_f_ *m*_f_/*N*_m_ *m*_m_≈21.6, respectively, for the two groups. For the bilineal agriculturalists (Figure 3D), the set of (*N*_f_/*N*, *m*_f_/*m*) values inferred from the 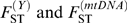 estimates fall within the range that was not rejected, given our data on X-linked and autosomal markers. For the patrilineal herders (Figure 3B), the overlap is only partial: from the NRY and mtDNA data only, low *N*_f_/*N* ratios associated with high *m*_f_/*m* ratios are as likely as high *N*_f_/*N* ratios associated with low *m*_f_/*m* ratios. Yet, it is clear from this figure that a large set of (*N*_f_/*N*, *m*_f_/*m*) values inferred from the single-locus estimates 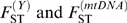 can be rejected, given the observed differentiation on X-linked and autosomal markers. All genetic systems (mtDNA, NRY, X-linked and autosomal markers) converge toward the notion that patrilineal herders, in contrast to bilineal agriculturalists, have a strong sex-specific genetic structure. Yet, the information brought by X-linked and autosomal markers is substantial, since we show that this is likely due to both higher migration rates and larger effective numbers for women than for men.

### Comparison with Other Studies

Our results, based on the X chromosome and the autosomes, also confirm previous analyses based on the mtDNA and the NRY, showing that men are genetically more structured than women in other patrilocal populations [3]–[10], [14]–[17] (see also Table 1). A handful of studies have also shown a reduced effective number of men compared to that of women, based on coalescent methods [23],[24], but none have considered the influence of social organization on this dissimilarity (see Table 1).

In some respects, our results contrast with those of Wilder and Hammer [25], who studied sex-specific population genetic structure among the Baining of New Britain, using mtDNA, NRY, and X-linked markers. Interestingly, they found that *N*_f_>*N*_m_, but *m*_f_<*m*_m_, and claimed that a similar result, although left unexplored by the authors, was to be found in a recent study by Hamilton et al. [16]. This raises the interesting point that sex-specific proportions of migrants (*m*) are likely to be shaped by factors that may only partially overlap with those that affect the sex-specific effective numbers (*N*). Further studies of human populations with contrasted social organizations, as well as further theoretical developments, are needed to appreciate this point.

In order to ask to what extent our results generalize to other human populations, we investigated sex-specific patterns in the 51 worldwide populations represented in the HGDP-CEPH Human Genome Diversity Cell Line Panel dataset [43], for which the data on the differentiation of 784 autosomal microsatellites and 36 X-linked microsatellites are available (data not shown). By doing this, we found a larger differentiation for X-linked than for autosomal markers 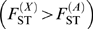. Therefore, we confirmed Ramachandran et al.'s [20] result that no major differences in demographic parameters between males and females are required to explain the X-chromosomal and autosomal results in this worldwide sample. Ramachandran et al.'s approach [20] is based upon a pure divergence model from a single ancestral population, which is very different from the migration-drift equilibrium model considered here. In real populations, however, genetic differentiation almost certainly arises both through divergence and limited dispersal, which places these two models at two ends of a continuum. Yet, importantly, if we apply Ramachandran et al.'s [20] model to the Central Asian data, our conclusions are left unchanged. In their model, the differentiation among populations is 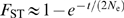, where *t* is the time since divergence from an ancestral population and *N*_e_ the effective size of the populations (see, e.g., [44]). Hence, we get 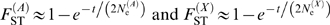 for autosomal and X-linked markers, respectively. Therefore, our observation that 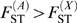 implies that 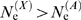, which requires that *N*_f_>7*N*_m_ since 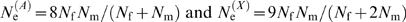 (see, e.g., [45]). In this case, the female fraction of effective number is larger than that of males, which is consistent with our findings in a model with migration.

The HGDP-CEPH dataset does not provide any detailed ethnic information for the sampled groups, and we can therefore not distinguish populations with different lifestyles. However, at a more local scale in Pakistan, we were able to analyze a subset of 5 populations (Brahui, Balochi, Makrani, Sindhi and Pathan), which are presumed to be patrilineal [46]. For this subset, we found a higher differentiation for autosomal 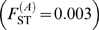 than for X-linked markers 
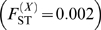
, although non-significantly (*p* = 0.12). This result seems to suggest that other patrilineal populations may behave like the Central Asian sample presented here. Therefore, because the geographical clustering of populations with potentially different lifestyles may minimize the differences in sex-specific demography at a global scale [21],[22], and/or because the global structure may reflect ancient (pre-agricultural) marital residence patterns with less pronounced patrilocality [12], we emphasize the point that large-scale studies may not be relevant to detect sex-specific patterns, which supports a claim made by many authors.

### Conclusion

In conclusion, we have shown here that the joint analysis of autosomal and X-linked polymorphic markers provides an efficient tool to infer sex-specific demography and history in human populations, as suggested recently [12],[47]. This new multilocus approach is, to our knowledge, the first attempt to combine the information contained in mtDNA, NRY, X-linked and autosomal markers (see Table 1), which allowed us to test for the robustness of a sex-specific genetic structure at a local scale. Unraveling the respective influence of migration and drift upon neutral genetic structure is a long-standing quest in population genetics [48],[49]. Here, our analysis allowed us to show that differences in sex-specific migration rates may not be the only cause of contrasted male and female differentiation in humans and that, contrary to the conclusion of a number of studies (see Table 1), differences in effective numbers may also play an important role. Indeed, we have demonstrated that sex-specific differences in population structure in patrilineal herders may be the consequence of both higher female effective numbers and female effective dispersal. Our results also illustrate the importance of analyzing human populations at a local scale, rather than global or even continental scale [2],[19],[21]. The originality of our approach lies in the comparison of identified ethnic groups that differ in well-known social structures and lifestyles. In that respect, our study is among the very few which compare patrilineal vs. bilineal or matrilineal groups (see Table 1), and we believe that it contributes to the growing body of evidence showing that social organization and lifestyle have a strong impact on the distribution of genetic variation in human populations. Moreover, our approach could also be applied on a wide range of animal species with contrasted social organizations. Therefore, we expect our results to stimulate research on the comparison of X-linked and autosomal data to disentangle sex-specific demography.

## Methods

### DNA Samples

We sampled 10 populations of bilineal agriculturalists and 11 populations of patrilineal herders from West Uzbekistan to East Kyrgyzstan, representing 780 healthy adult men from 5 ethnic groups (Tajiks, Kyrgyz, Karakalpaks, Kazaks, and Turkmen) (see Table 2). The geographic distribution of the samples and information about lifestyle is provided in Figure 1. Also living in Central Asia, Uzbeks are traditionally patrilineal herders too, but they have recently lost their traditional social organization [11], and we therefore chose not to include any sample from this ethnic group for the purpose of this study. We collected ethnologic data prior to sampling, including the recent genealogy of the participants. Using this information, we retained only those individuals that were unrelated for at least two generations back in time. All individuals gave their informed consent for participation in this study. Total genomic DNA was isolated from blood samples by a standard phenol-chloroform extraction [50].

### Uniparentally Inherited Markers

The mtDNA first hypervariable segment of the mtDNA control region (HVS-I) was amplified using primers L15987 (5′TCAAATGGGCCTGTCCTTGTA) and H580 (5′TTGAGGAGGTAAGCTACATA) in 18 populations out of 21 (674 individuals, see Table 2). The amplification products were subsequently purified with the EXOSAP standard procedure. The sequence reaction was performed using primers L15925 (5′TAATACACCAGTCTTGTAAAC) and HH23 (5′AATAGGGTGATAGACCTGTG). Sequences from positions 16 024–16 391 were obtained. Eleven Y-linked microsatellite markers (see Table 3) were genotyped in the same individuals, following the protocol described by Parkin et al. [51].

### Multi-Locus Markers

27 autosomal and 9 X-linked microsatellite markers (see Table 4) were genotyped in the same individuals. We used the informativeness for assignment index *I*_n_ [52] to select subsets of microsatellite markers on the X chromosome and the autosomes from the set of markers used in Rosenberg et al.'s worldwide study [43]. This statistic measures the amount of information that multiallelic markers provide about individual ancestry [52]. This index was calculated among a subset of 14 populations, chosen from the Rosenberg et al.'s dataset [43] to be genetically the closest to the Central Asian populations (Balochi, Brahui, Burusho, Hazara, Pathan, Shindi, Uygur, Han, Mongola, Yakut, Adygei, Russian, Druze and Palestinian). The rationale was to infer the information provided by individual loci about ancestry from this subset of populations, and to extrapolate the results to the populations studied here. For the X chromosome data, we pooled the ‘Screening Set10’ and ‘Screening Set52’ from the HGDP-CEPH Human Genome Diversity Cell Line Panel [53] analyzed by Rosenberg et al. [43] which represented a total of 36 microsatellites. We chose 9 markers among the 11 with the highest *I*_n_. For autosomal data, we used the ‘Screening Set10’, which represented a total of 377 microsatellites, and chose 27 markers among the 30 with the highest *I*_n_. All markers were chosen at a minimum of 2 cM apart from each others [54]. PCR amplifications were performed in a 20 µl final volume composed of 1× Eppendorf buffer, 125 µM each dNTP, 0.5U Eppendorf Taq polymerase, 125 nM of each primer, and 10 ng DNA. The reactions were performed in a Eppendorf Mastercycler with an initial denaturation step at 94°C for 5 min; followed by 36 cycles at 94°C for 30 s, 55°C for 30 s, 72°C for 20 s, and 72°C for 10 min as final extension. Forward primers were fluorescently labeled and reactions were further analyzed by capillary electrophoresis (ABI 310, Applied Biosystems). We used the software package Genemarker (SoftGenetics LLC) to obtain allele sizes from the analysis of PCR products (allele calling).

### Statistical Analyses

We calculated the total allelic richness (*AR*) (over all populations), the unbiased estimate of expected heterozygosity *H*_e_ [55], the total number of polymorphic sites and *F*_ST_ for mtDNA using Arlequin version 3.1. [56]. Genetic differentiation among populations for the autosomes, the X and the Y chromosome was measured both per locus and overall loci using Weir and Cockerham's *F*_ST_ estimator [57], as calculated in Genepop 4.0. [58]. The 95% confidence intervals were obtained by bootstrapping over loci [58], using the approximate bootstrap confidence intervals (ABC) method described by DiCiccio and Efron [59]. Isolation by distance (i.e. the correlation between the genetic and the geographic distances) was analyzed by computing the regression of pairwise *F*_ST_/(1−*F*_ST_) estimates between pairs of populations to the natural logarithm of their geographical distances, and rank correlations were tested using the Mantel permutation procedure [60], as implemented in Genepop 4.0. [58]. All other statistical tests were performed using the software package R v. 2.2.1 [61].

### Sex-Biased Dispersal in the Island Model

Let us consider an infinite island model of population structure [62], with two classes of individuals (males and females), which describes a infinite set of populations with constant and equal sizes that are connected by gene flow. Then the expected values of *F*_ST_ for uniparentally inherited markers depend on the effective number *N*_m_ (resp. *N*_f_) of adult males (resp. females) per population and the migration rate *m*_m_ (resp. *m*_f_) of males (resp. females) per generation, as: 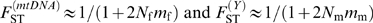 (see, e.g., [63]). We can therefore calculate the female-to-male ratio of the effective number of migrants per generation as: 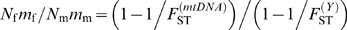.

In this model, we can also compute for the autosomes and the X chromosome the reproductive values for each class (sex), which are interpreted here as the probability that an ancestral gene lineage was in a given class in a distant past [64]. From these, we can obtain the well-known expressions of effective size *N*_e_ for autosomal and X-linked genes: 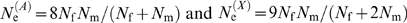, respectively [45]. Note that *N*_e_ is expressed here as a number of gene copies (i.e., twice the effective number of diploid individuals for autosomes). Likewise, the effective migration rate, i.e. the average dispersal rate of an ancestral gene lineage, is given by 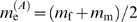 for autosomal genes, and 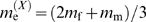 for X-linked genes, respectively. Substituting these expressions into the well-known equation: *F*_ST_≈1/(1+2*N*_e_ *m*_e_) [64], we get:
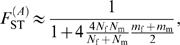
(5)for autosomal genes, and
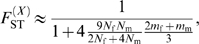
(6)for X-linked genes.

### Evaluation of the Approach through Stochastic Simulations

We performed coalescent simulations, using an algorithm in which coalescence and migration events are considered generation-by-generation until the common ancestor of the whole sample has been reached (see [65]). We simulated a finite island model with 50 demes, each made of *N* = *N*_f_+*N*_m_ = 500 diploid individuals, with a migration parameter *m* = *m*_f_+*m*_m_ = 0.2. Using these total values for *N* and *m*, we then varied the sex-specific parameters to cover the (*N*_f_/*N*, *m*_f_/*m*) parameter space evenly. Note that the parameter *m* is the total migration rate, which corresponds to twice the effective migration rate for autosomal markers. Hence, for each set of (*N*_f_/*N*, *m*_f_/*m*) values, the total number of individuals is 500 (although the number of females may vary from 1 to 499) and the effective migration rate for autosomal markers is 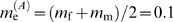. We chose these total values for *N* and *m* such that, for a ratio *N*_f_ *m*_f_/*N*_m_ *m*_m_ = 21.6 (as observed for the herder populations), the distribution of *F*_ST_ estimates on uniparentally-inherited markers in the simulations were close to the observations: for mtDNA, the 95% highest posterior density interval (see [66], pp. 38–39) for the distribution of *F*_ST_ estimates in the simulations was [0.007; 0.033] with a mode at 0.014 (estimated value from the real dataset: 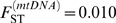 among the herders) while for the NRY, the 95% highest posterior density interval was [0.088; 0.374] with a mode at 0.187 (estimated value from the real dataset: 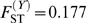).

Each simulated sample consisted in 330 sampled males from 11 populations (30 males per population), genotyped at 27 autosomal, 9 X-linked markers as well as 10 Y-linked markers and a single mtDNA locus. Each locus was assumed to follow a Generalized Stepwise Model (GSM) [67] with a possible range of 40 contiguous allelic states, except the mtDNA, which was assumed to follow an infinite allele model of mutation. The average mutation rate was 5.10^−3^, and the mean parameter of the geometric distribution of the mutation step lengths for microsatellites was set to 0.2 [67],[68].
